# Beyond Acquired Von Willebrand Deficiency: Exploring Alternative Mechanisms of Heyde's Syndrome

**DOI:** 10.7759/cureus.41494

**Published:** 2023-07-07

**Authors:** Alvin Oommen, Kripali Gautam, Akash Kumar

**Affiliations:** 1 Internal Medicine, Montefiore Medical Center, New York, USA; 2 Internal Medicine, New York University (NYU) Langone Health, New York, USA; 3 Gastroenterology, Montefiore Medical Center, New York, USA

**Keywords:** transcutaneous aortic valve replacement, acquired von willebrand syndrome, heyde's syndrome, gastrointestinal bleed, aortic stenosis, intestinal angiodysplasia

## Abstract

Heyde's syndrome (HS) is a complex condition characterized by the coexistence of severe aortic stenosis (AS) and gastrointestinal (GI) angiodysplasia. The prevailing belief has been that acquired von-Willebrand factor deficiency (AVWD) is the underlying cause of HS. However, the validity of this theory remains contentious, as there have been reports of bleeding angiodysplasia in the setting of AS despite normal von-Willebrand factor (vWF) activity. Here, we present a compelling case of HS with negative diagnostic testing for AVWD. A 61-year-old female with a history of end-stage renal disease on hemodialysis, AS, and a history of recurrent GI bleeding presented with dyspnea. Prior to arrival, she reported multiple episodes of melena and hematochezia and was found to have a hemoglobin of 6 g/dL. Notable exam findings included melenic stool on digital rectal exam and a grade three systolic crescendo-decrescendo murmur that radiated up to the carotids. A transthoracic echocardiogram demonstrated evidence of severe AS. Considering the recurrent GI bleeding and severe AS, HS was suspected. To investigate this further, a vWF disease panel was sent, revealing a normal multimeric pattern. Given hemodynamic stability, she was discharged but had multiple readmissions soon after with recurrent GI bleeding requiring endoscopic intervention. On her last visit, she underwent transcatheter aortic valve replacement (TAVR) with notable resolution in her GI bleeds thereafter. The prevailing theory regarding the etiology of HS is acquired vWF deficiency. However, the validity of this theory remains a topic of debate, as a growing body of evidence suggests that the absence of AVWD does not necessarily rule out the diagnosis. The absence of AVWD in our patient raises questions about its prevalence in HS and its status as a key feature and highlights the importance of considering HS events without AVWD, given the risk of recurrent life-threatening GI bleeds.

## Introduction

Heyde’s syndrome (HS) is a rare condition characterized by aortic stenosis (AS) and gastrointestinal (GI) bleeding often due to angiodysplasia that was first described by Edward Heyde in 1958 [1].  It is primarily diagnosed in the older adult population, typically in patients aged 65 years or older. Limited information exists regarding the exact prevalence of HS. However, considering the common occurrence of GI bleeding and AS in the general population, both of which increase with age, HS is likely to be underreported and underdiagnosed. Currently, the most accepted mechanism of HS suggests that increased shear stress forces from moderate-severe AS induces conformational changes in von Willebrand factor (vWF) multimers, leading to acquired Von Willebrand disease (AVWD) [2]. However, the validity of this theory remains contentious, as there have been reports of bleeding angiodysplasia in the setting of AS despite normal vWF activities. Furthermore, numerous cases of HS reported in the existing literature lack supporting evidence for AVWD, often foregoing appropriate testing for the condition [3]. We present a compelling case of HS characterized by severe AS and GI bleeding from angiodysplasia with negative diagnostic testing for AVWD.

## Case presentation

A 61-year-old woman with a history of end-stage renal disease (ESRD) on hemodialysis (HD), hypertension, type 2 diabetes, atrial fibrillation, coronary artery disease with stent placement, AS, and a history of recurrent GI bleeding, presented with dyspnea and decreased exercise tolerance. Of note, the patient experienced multiple episodes of GI bleeding a year prior to her current presentation. Endoscopic evaluation revealed angioectasia, ultimately requiring her to stop anticoagulation, nonsteroidal anti-inflammatory drugs (NSAIDs), and platelet therapy at the time.

Prior to arrival, she reported experiencing multiple episodes of melena at home and was sent to the emergency department from HD after being found to have a hemoglobin of 6 g/dL. On arrival, she was hemodynamically stable with melenic stool noted on the digital rectal exam and a grade three systolic crescendo-decrescendo murmur that radiated up to the carotids. With the exception of an elevated creatinine of 3.35 mg/dL, consistent with her ESRD, the rest of her lab values were within normal limits. She was transfused with two units of packed red blood cells (PRBCs) and admitted to the general medicine floor.

The patient experienced multiple episodes of melena throughout her admission, necessitating several blood transfusions. During the course of her evaluation, she underwent an esophagogastroduodenoscopy (EGD), which revealed a non-bleeding gastric clean-based ulcer, which was deemed unlikely to be the source of ongoing bleeding. Subsequently, a video capsule endoscopy (VCE) was performed, uncovering discrete areas of fresh blood in the distal duodenum and an isolated site of fresh blood in the mid small bowel. Despite a subsequent push enteroscopy, the source of bleeding could not be identified.

A transthoracic echocardiogram (TTE) demonstrated a mildly decreased left ventricular ejection fraction (LVEF) of 45% and severe AS with an aortic valve area of 0.9 cm2. Considering the recurrent GI bleeding and severe AS, HS was suspected. To investigate this further, a vWF disease panel was conducted, showing normal levels of vWF antigen (153%), ristocetin cofactor activity (101%), and factor VIII coagulation assay (191%). In addition, vWF multimeric analysis revealed a normal multimeric pattern. With the patient being hemodynamically stable, she was discharged with scheduled follow-up appointments with gastroenterology and structural cardiology. 

**Figure 1:**
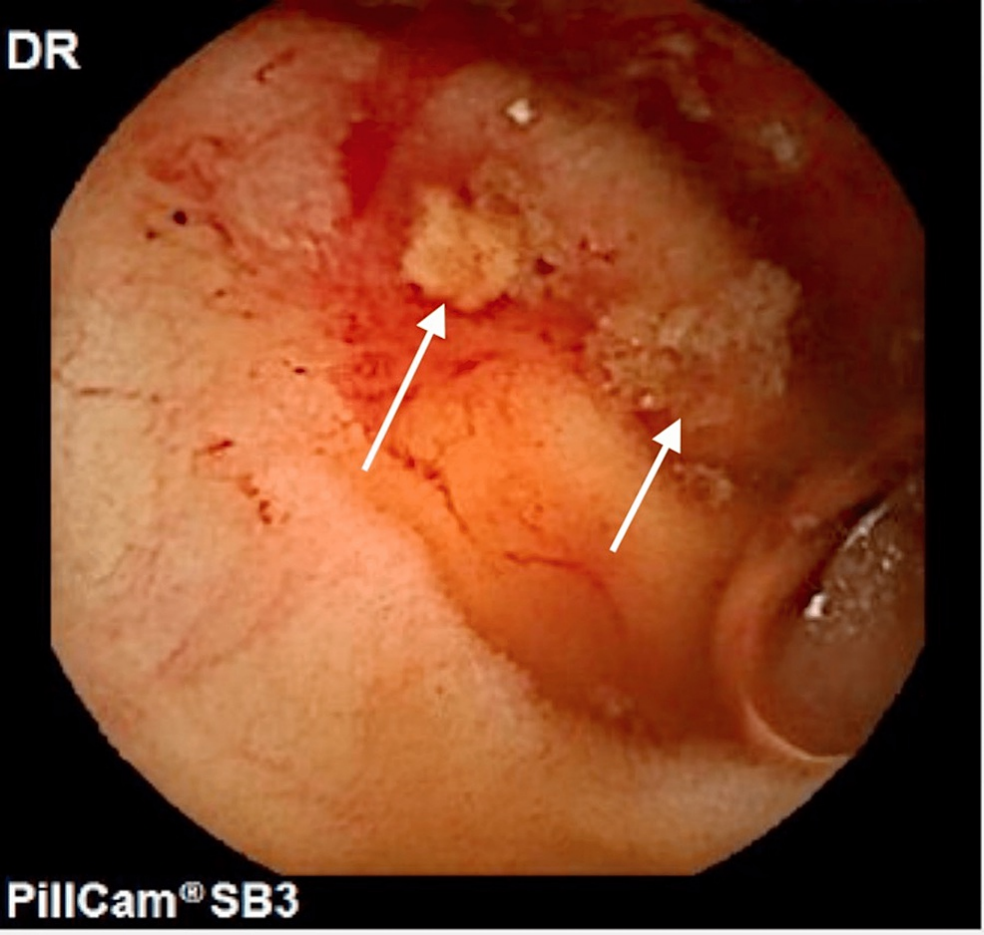
Video capsule endoscopy revealing small ulcers indicated by white arrows in the distal small intestine.

**Figure 2:**
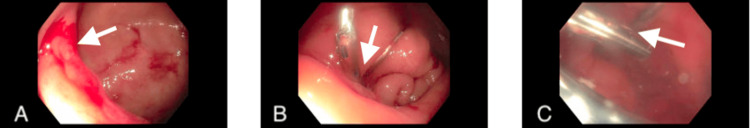
Colonic angioectasia (A) indicated by white arrow, successfully treated with hemoclips (B, C).

**Figure 3:**
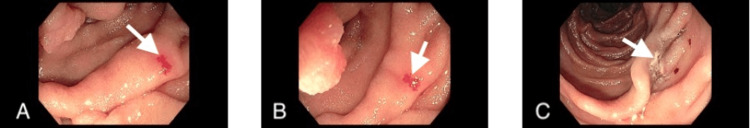
Angiodysplastic lesion in the fourth portion of the duodenum indicated by the white arrow (A, B), successfully treated with APC (C). APC: argon plasma coagulation

**Figure 4:**
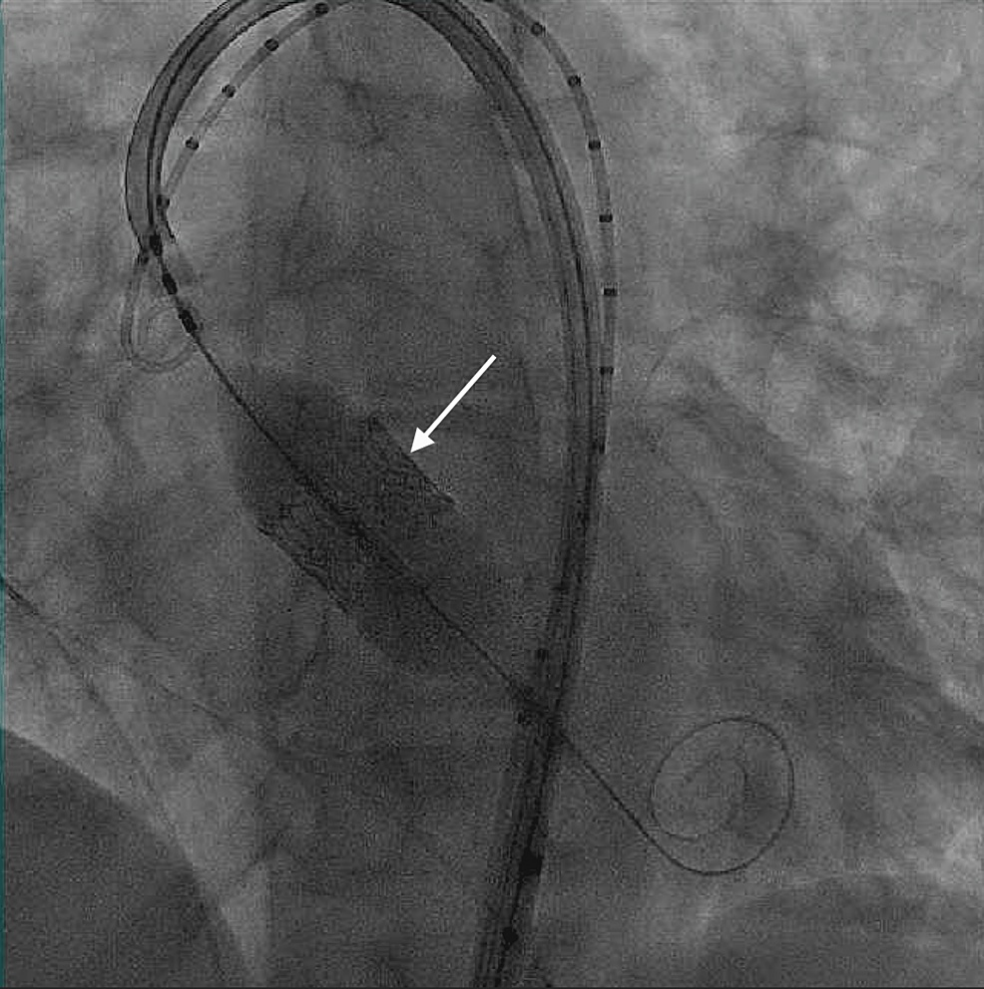
Intraprocedural fluoroscopy revealing appropriately expanded replacement aortic valve indicated by the white arrow.

A month later, she once again sought medical attention due to recurrent GI bleeding and symptomatic anemia. Repeat VCE revealed several small ulcers oozing blood in the distal small intestine (Figure 1). Colonoscopy also noted blood in the entire examined colon with a single bleeding colonic angioectasia (Figure 2A-2C) that was successfully treated with hemoclips. She returned once again a week later due to recurrent GI bleeding. Push enteroscopy identified a single angiodysplastic lesion without active bleeding in the fourth portion of the duodenum (Figure 3A-3C), which was successfully treated using argon plasma coagulation (APC). Given multiple admissions for GI bleeding, the repair of her AS was deemed necessary, and she underwent transcatheter aortic valve replacement (TAVR) with no complication (Figure 4). Her hemoglobin remained stable throughout the remainder of her hospitalization, and she was discharged with no further readmissions or follow-up visits related to GI bleeding.

## Discussion

HS is a complex condition that is characterized by severe aortic valve stenosis and angiodysplasia in the GI tract. The prevailing theory regarding the etiology of this syndrome is acquired vWF deficiency [4]. According to this presumed pathophysiology, the shear stress exerted by severely stenotic aortic valves leads to conformation changes in vWF, making it more susceptible to cleavage by ADAMTS13. This can subsequently lead to the development of von Willebrand syndrome type 2A, a coagulopathy that predisposes patients to angiodysplasia [4]. This hypothesis was more widely accepted after Vincentelli et al. found that vWF abnormalities in patients with AS are directly related to the severity of AS and improve with valve replacement [5].

However, the validity of this theory remains a topic of debate, as a growing body of evidence suggests that the absence of acquired von Willebrand syndrome does not necessarily rule out the diagnosis [3]. This notion is illustrated by the present case, which describes a patient with characteristics of HS, where the vWF panel and multimeric analysis did not indicate AVWD. Nevertheless, the patient's recurrent episodes of GI bleeding in the presence of severe AS despite intervention was characteristic of the syndrome. Subsequent identification of colonic and duodenal angioectasia during push enteroscopy and colonoscopy, which resolved only after TAVR, provided even stronger evidence for the diagnosis. Furthermore, while AVWD is considered the most likely etiology of HS, a surprising amount of the current literature reporting HS does not include confirmatory testing. A recent systematic review found that 50/77 (64.9%) of reported cases of HS provides no explicit evidence of AVWD [3]. Furthermore, similar to our patient, in 18% of the cases that did conduct testing, AVWD was negative, which raises the suspicion of alternative mechanisms behind the syndrome.

Several alternative theories have been suggested, one of which suggests increased angiogenesis in the gut as mucosal hypoperfusion can cause decreased cardiac output through a severely stenosed aortic valve [6]. Another postulates that the alteration of pulse waveforms from the AS can result in angiodysplasia, similar to hypertrophic cardiomyopathy [7]. It has also been suggested that low-grade chronic hypoxia may stimulate reflex vasodilation of vasculature of the gut, thereby leading to angioectasia [8]. Regardless, the absence of AVWD in our patient raises important questions about the prevalence of this condition in HS and whether it should be considered a key feature of the syndrome.

## Conclusions

HS is a complex multisystem disorder that at times can present as a diagnostic challenge. While AVWD has been traditionally considered as the prevailing etiology, the presence of AVWD has not been consistently demonstrated in the literature. This raises questions about the existence of alternative pathways contributing to HS. It also highlights the importance of considering HS even in patients without AVWD, especially given the increased risk of recurrent life-threatening GI bleeding without aortic valve replacement. Further investigation is needed to elucidate these alternative mechanisms, improve diagnostic capabilities, and optimize the management of HS in clinical practice.
